# Effects of 3′-isovaleryl-4′-senecioylkhellactone from *Peucedanum japonicum* Thunberg on PMA-Stimulated Inflammatory Response in A549 Human Lung Epithelial Cells

**DOI:** 10.4014/jmb.2107.07001

**Published:** 2021-11-14

**Authors:** Daseul Hwang, Hyung Won Ryu, Ji-Won Park, Jung-Hee Kim, Doo-Young Kim, Jae-Hoon Oh, Ok-Kyoung Kwon, Sang-Bae Han, Kyung-Seop Ahn

**Affiliations:** 1 Natural Medicine Research Center, Korea Research Institute of Bioscience and Biotechnology, Cheongju 28116, Republic of Korea; 2 College of Pharmacy, Chungbuk National University, Cheongju 28160, Republic of Korea

**Keywords:** 3′-isovaleryl-4′-senecioylkhellactone, *Peucedanum japonicum* Thunberg, inflammation, NF-κB, AP-1

## Abstract

*Peucedanum japonicum* Thunberg (PJT) has been used in traditional medicine to treat colds, coughs, fevers, and other inflammatory diseases. The goal of this study was to investigate whether 3′-isovaleryl-4′-senecioylkhellactone (IVSK) from PJT has anti-inflammatory effects on lung epithelial cells. The anti-inflammatory effects of IVSK were evaluated using phorbol 12-myristate 13-acetate (PMA)-stimulated A549 cells and regular human lung epithelial cells as a reference. IVSK reduced the secretion of the inflammatory mediators interleukin (IL)-8 and monocyte chemoattractant protein-1 (MCP-1), and the mRNA expression of IL-6, IL-8, MCP-1, and IL-1β. Additionally, it inhibited the phosphorylation of IκB kinase (IKK), p65, Iκ-Bα, and mitogen-activated protein kinases (MAPKs) p38, JNK, and ERK in A549 cells stimulated with PMA. Moreover, the binding affinity of activator protein-1 (AP-1) and nuclear factor-κB (NF-κB) was significantly reduced in the luciferase assay, while nuclear translocation was markedly inhibited by IVSK in the immunocytochemistry. These findings indicate that IVSK can protect against inflammation through the AP-1 and NF-κB pathway and could possibly be used as a lead compound for the treatment of inflammatory lung diseases.

## Introduction

Inflammation is a protective response against harmful stimuli and nonspecific reaction mediated by innate immunity. The purpose of inflammation is to inhibit cell damage, remove destroyed tissue and necrotic cells from the wound, and regenerate tissue at the same time. Inflammatory abnormalities in the lungs underlie a vast variety of diseases such as asthma, acute lung injury (ALI) and sepsis which are caused by the release of inflammatory mediators as well as the infiltration and activation of immune cells in lung [1, 2].

In asthma, macrophages become activated when allergen attaches to a FcεRII receptor on the surface of the macrophages [3]. These activated macrophages secrete IL-6 to stimulate lung epithelial cells, which in turn secrete IL-8 to induce inflammation [4]. Additionally, the epithelial cells, macrophages, and lymphocytes produce monocyte chemoattractant protein-1 (MCP-1) [5], which moves eosinophils from the blood vessels to the airways. The eosinophils accumulated in the airways lead to airway obstruction [6]. In addition, IL-1β and IL-6 that are released from the epithelial cells and macrophages, respectively, also amplify the inflammatory response [7]. More specifically, IL-1β activates NF-κB and AP-1, both of which have important roles in the exacerbation of lung inflammation [8, 9].

NF-κB is an inducible transcriptional factor for the regulation of numerous inflammatory proteins, and it also increases allergic inflammation of the airways, mucus secretion and airway hypersensitivity, which lead to symptoms of asthma in particular. Therefore, inhibition of NF-κB in lung epithelial cells can be a means of reducing the severity of lung inflammation [10, 11]. AP-1 is a protein transcription factor derived from two superfamilies, JUN and FOS, and generally functions as a positive factor in regulating inflammation and cell cycle. However, it increases the inflammatory response in cooperation with other transcription factors such as NF-κB. Inhibition of the AP-1 activity in the inflammatory response not only increases anti-inflammatory cytokines such as IL-10 but also downregulates inflammatory cytokines such as MCP-1 and IL-8 [8, 12]. MAPK is involved in several processes such as immune and inflammatory reactions and tumorigenesis in response to various extracellular and intracellular stimuli. In particular, the IKK complex phosphorylates p105 to induce degradation and activate ERK1/2, effectively. Activation of IKK and MAPK activates both NF-κB and AP-1 [13].

Asthma affected around 300 million people worldwide in 2019, and patients with asthma mostly use anti-leukotriene agents or corticosteroid to control their symptoms [14]. However, since long-term use of steroids may cause side effects such as thrush, cataracts, and osteopenia [15], the development of a new therapeutic agent with fewer side effects is required.

**Fig. 1:**
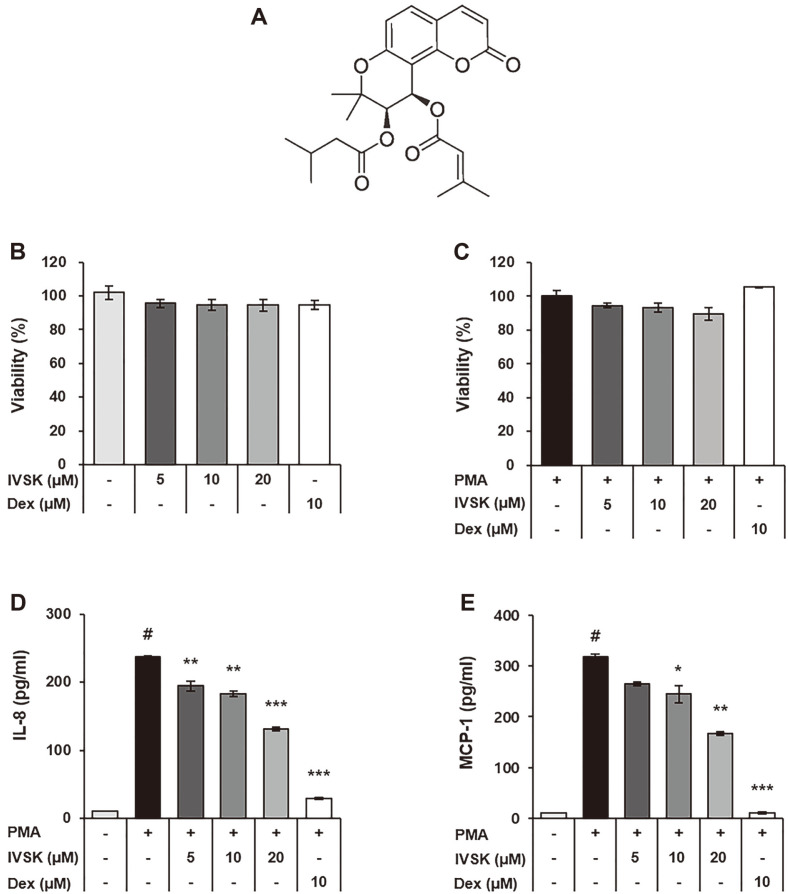
Effects of IVSK on cell viability and inflammatory mediators in PMA-stimulated A549 cells. The structure of 3′-isovaleryl-4′-senecioylkhellactone (**A**). A549 cells were treated with IVSK at each indicated concentration for 24 h (**B**) and with 10 nM PMA (**C**). The viability was measured by the CytoX assay. A549 cells were treated with IVSK at each indicated concentration and subsequently stimulated with 10 nM PMA. The expression of IL-8 (**D**) and MCP-1 (**E**) was measured by ELISA. Data are expressed as the mean ± SD. ^#^*p* < 0.05 vs. negative control group; *p < 0.05, **p < 0.01 and ***p < 0.001 vs. PMA only group. Dex: Dexamethasone.

*Peucedanum japonicum* Thunberg (PJT) has been used in traditional medicine to treat colds, gout, coughs, headaches, fever and other inflammatory diseases [16]. It is native to north central and southeastern China, Japan and Korea, and is widely cultivated [17]. A recent study has shown that extracts of PJT are also effective against asthma by inhibiting ovalbumin (OVA)-induced allergies and by downregulating interleukin (IL)-5, IL-6, IL-13, inducible nitric oxide synthase (iNOS), and cyclooxygenase-2 (COX-2) [18]. Among the various constituent compounds found in PJT, 3'-isovaleryl-4'-senecioylkhellactone (IVSK; Fig. 1A) was reported to have an IC_50_ value of 9.9 ± 0.08 μM by nitric oxide (NO) assay, which is lower than that of aminoguanidine, a positive control, at 16.9± 0.12 μM [19].

However, to the best of our knowledge, no detailed mechanistic studies on the anti-inflammatory effects of IVSK in lung inflammation have ever been conducted. We therefore addressed herein the evidence describing the effects of IVSK on inflammatory markers produced via IKK/NF-κB and MAPK/AP-1 pathway in PMA-induced epithelial cells.

## Materials and Methods 

### Preparation of Compound

PJT was collected from Jeju Island, South Korea, in June 2012 and identified by Dr. Joong-Ku Lee. A voucher specimen recorded as PBC-484 was deposited at the Korea Plant Extract Bank. Won *et al*. [20] purified IVSK (38.0 mg, purity > 99%) from *P. japonicum* roots and identified it using UPLC-MS-based separation. A stock solution (20 mM) was prepared in DMSO. IVSK, provided by Won *et al*., was purified and identified.

### Cell Culture

The A549 human lung epithelial cell line from the American Type Culture Collection (USA) was cultured in RPMI 1640 (Cat. No. LM 011-01, Welgene, Korea) containing 10% FBS (Cat. No. S001-07, Welgene) and 1%penicillin-streptomycin (Cat. No. 15070063, Thermo Fisher Scientific Inc., USA). The cells were incubated at 37°C in a 5% CO_2_ incubator.

### Cell Viability Assay

The cell viability was measured by the CytoX Assay Kit (Cat. No. CYT3000, LPS Solution, Korea). The A549 cells were seeded in a 96-well plate and treated with 5, 10, and 20 μM of IVSK for 20 h. The CytoX solution was added to the well and then incubated for 4 h. The absorbance was measured at 450 nm by the Spark 10M Multimode microplate reader (Tecan Inc., Switzerland).

### Firefly Luciferase Reporter Assay

A549 cells were transfected using the LipofectAMINE 3000 reagent (Invitrogen, USA) with the NF-κB pGL4.32 vector (Promega, USA) and AP-1 pGL4.44 vector (Promega). The transfected cells were treated with IVSK for 1 h and stimulated with 100 and 50 nM phorbol 12-myristate 13-acetate (PMA, USA) for 16 h. The cell lysates were examined with the luciferase reporter assay system (Promega). The luciferase activity was measured by the Spark 10M Multimode microplate reader. The luciferase activity was normalized to *Renilla* luciferase activity to determine the transfection efficiency.

### Enzyme-Linked Immunosorbent Assay (ELISA)

The A549 cells were seeded in a 60 mm dish at 1.2 × 10^6^ cells/dish and treated with IVSK (5-20 μM) for 1 h before being stimulated with PMA (10 nM). Six hours after the PMA treatment, the supernatant was harvested. The cytokine and chemokine levels were measured using IL-8 and MCP-1 ELISA kits (IL-8 set, Cat. No. DY208; MCP-1 set, Cat. No. DY279; R&D Systems Inc., USA) [21]. The absorbance was detected at 450 nm by the Spark 10M Multimode microplate reader.

### RT-PCR Analysis

RNA was isolated from A549 cells using the TRIzol reagent (Cat. No. 15596018, Invitrogen). The A549 cells were seeded in a 6-well plate at 2.5 × 10^5^ cells/ml and treated with various concentrations of IVSK (5-20 μM) for 1 h. After that, the cells were treated with 10 nM PMA for 5 h. The purity of the RNA was measured using a spectrophotometer (NanoDrop 2000c; Thermo). Reverse transcription was done with a cDNA synthesis kit (Qiagen, Germany). The cDNA was amplified by PCR using primers (Table 1). The PCR products were separated on a 1.5% agarose gel with the RedSafe™ Kit (Intron Biotechnology, Inc., Korea). The gel image was captured using the Olympus C4000 camera system (Olympus, Japan) and analyzed by ImageJ software (version 1.50e; National Institutes of Health, USA). Band intensity was normalized to GAPDH.

### Western Blotting

The A549 cells were seeded in a 60 mm dish at 1.2 × 10^6^ cells/dish and treated with various concentrations of IVSK (5-20 μM) for 1 h. After that, the cells were treated with 10 nM PMA for 1 h before the cells were harvested. Proteins were prepared with NP-40 buffer (Cat. No. EBA-1049, ELPIS-Biotech Inc., Korea) containing protease and phosphatase or the NE-PER Nuclear and Cytoplasmic Extraction Reagents (Cat. No, 78833, Thermo). Protein extracts were measured by the Pierce BCA Protein Assay Kit (Cat. No. 23225, Thermo) to quantify the extracted proteins. Proteins were separated by 10% SDS-PAGE and transferred to PVDF membranes (EMD Millipore, USA). The membranes were incubated in 5% skim milk in Tris-buffered saline-Tween 20 (TBS-T) buffer at room temperature for 1 h and then incubated with primary antibodies with 5% skim milk in TBS-T at 4°C overnight: IKK (Cat. No. sc-8943, Santa Cruz Biotechnology, USA), p-IKK (Cat. No. 2697, Cell Signaling Technology, USA), p65 (Cat. No. 8242, CST), p-p65 (Cat. No. 3033, CST), IκBα (Cat. No, MA5-15132, Invitrogen), p-IκBα (Cat. No. MA5-15087, Invitrogen), β-actin (Cat. No. sc-69879, Santa Cruz), Lamin A/C (Cat. No. sc-6215, Santa Cruz), c-Fos (Cat. No. 2250, CST) and c-Jun (Cat. No. 9165, CST). After washing, the secondary antibody was incubated at room temperature for 1 h. The membranes were visualized by an enhanced chemiluminescence kit (ECL; Cat. No. 32106, Thermo) and the Amersham Imager 680 (GE Healthcare, USA).

### Immunocytochemistry

The A549 cells were seeded in a Nunc Lab-Tek II Chamber Slide System (Cat. No. 154534, Nunc) at 5 × 10^4^ cells/well and treated with 20 μM of IVSK for 1 h. After that, the cells were treated with 10 nM PMA for 1 h, then fixed in 10% Neutral Buffered Formalin (Cat. No. BBC 0150, BBC Biochemical, USA) for 15 min at room temperature, and washed 3 times with phosphate-buffered saline (PBS). A549 cells were permeabilized with 0.2% Triton X-100 in PBS for 15 min at 4°C, then washed 3 times with PBS, and blocked with 3% bovine serum albumin in PBS. Next, the cells were incubated with the primary antibodies NF-κB p65 (Cat. No. 8242, CST), c-Fos (Cat. No. 2250, CST), and c-Jun (Cat. No.9165, CST) at 4°C overnight. After incubation, the A549 cells were washed with PBS 5 times and incubated with anti-rabbit Alexa Fluor 488-conjugated secondary antibody at 4°C for 1 h and washed with PBS 5 times. And then, the cells were incubated with Hoechst 33342 nucleic acid stain (Cat. No. H3570, Thermo) for 5 min and mounted with coverslips for visualization with an Axio Observer Z1 microscope (Zeiss, Germany).

### Statistical Analysis

For the statistical analysis, each data point was expressed as the mean ± standard deviation (SD). Statistical significance was determined using the two-tailed Student’s *t*-test. *p*-values less than 0.05 were considered to indicate a statistically significant difference.

## Results 

### Effects of IVSK on the Cell Viability and Inflammatory Mediators of PMA-Stimulated A549 Cells

The cytotoxicity tests for IVSK showed that the viability of the A549 cells was not affected by IVSK for doses of up to 20 μM (Fig. 1B). Similar results were obtained in the presence of 10 nM PMA as well (Fig. 1C). Therefore, the maximum concentration of IVSK used in this study was 20 μM.

To confirm the anti-inflammatory effect of IVSK in A549 cells, the secretion of inflammatory cytokines and chemokines was confirmed by ELISA. When A549 cells were treated with PMA, the chemokines IL-8 and MCP-1 were increased due to inflammation. PMA activates neutrophils and monocytes and has an important role in recruiting immune cells to the inflammatory site. When A549 cells were treated only with PMA, we could confirm the increased secretion levels of IL-8 and MCP-1. However, in the presence of IVSK, both IL-8 (Fig. 1D) and MCP-1 (Fig. 1E) showed reduced levels of secretion with a further decreasing trend as more IVSK was introduced. Overall, the data support the claim that IVSK reduces the inflammatory response by inhibiting the secretion of pro-inflammatory mediators in PMA-stimulated A549 cells.

### Effects of IVSK on PMA-Stimulated Pro-Inflammatory Mediators at the mRNA Level in A549 cells

Because IVSK exhibited inhibitory effects on the secretion of inflammatory mediators in PMA-stimulated A549 cells in ELISA, RT-PCR was performed to examine the mRNA levels of the inflammatory mediators. Along with IL-8 and MCP-1, IL-6 and IL-1β are cytokines that amplify the inflammatory response when treated with PMA in A549 cells. These early inflammatory mediators can be directly or indirectly involved in the activation of transcription factors such as NF-κB and AP-1.

**Fig. 2:**
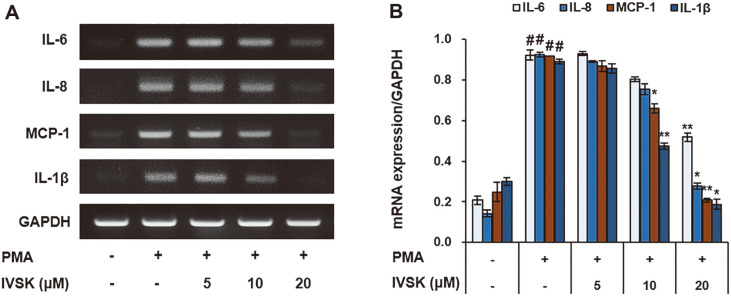
Effects of IVSK on the expression of pro-inflammatory mediators at the mRNA level in PMAstimulated A549 cells. A549 cells were treated with IVSK at each respective concentration and subsequently stimulated with 10 nM PMA. The RNA expression levels are shown for IL-6, IL-8, MCP-1 and IL-1β (**A**). GAPDH is an internal control for creating the graph (**B**). Data are expressed as the mean ± SD. ^#^*p* < 0.05 vs. negative control group; *p < 0.05, **p < 0.01 and ***p < 0.001 vs. PMA only group.

In a standard procedure, when A549 cells were treated only with PMA, it was confirmed that the production of IL-6, IL-8, MCP-1, and IL-1β increased. Upon the addition of IVSK, the levels of IL-6, IL-8, MCP-1, and IL-1β decreased, more so for the higher concentrations of IVSK used (Figs. 2A-2B). The results of the RT-PCR clearly show that IVSK would inhibit the expression of pro-inflammatory factors. In particular, IL-8 and MCP-1 showed a significant decrease in the RT-PCR results similar to the ELISA results.

### Effects of IVSK on the Phosphorylation of IKK and NF-κB and the Translocation of p65 in PMA-Stimulated A549 Cells

It was confirmed that the prevalence of pro-inflammatory mediators decreased with the addition of IVSK, as shown by the ELISA and RT-PCR results. Therefore, luciferase assays and western blots were performed to examine the degree of gene expression of NF-κB p65 in PMA-stimulated A549 cells. NF-κB p65 has an important role in the immune response inducing inflammatory mediators [22].

**Fig. 3:**
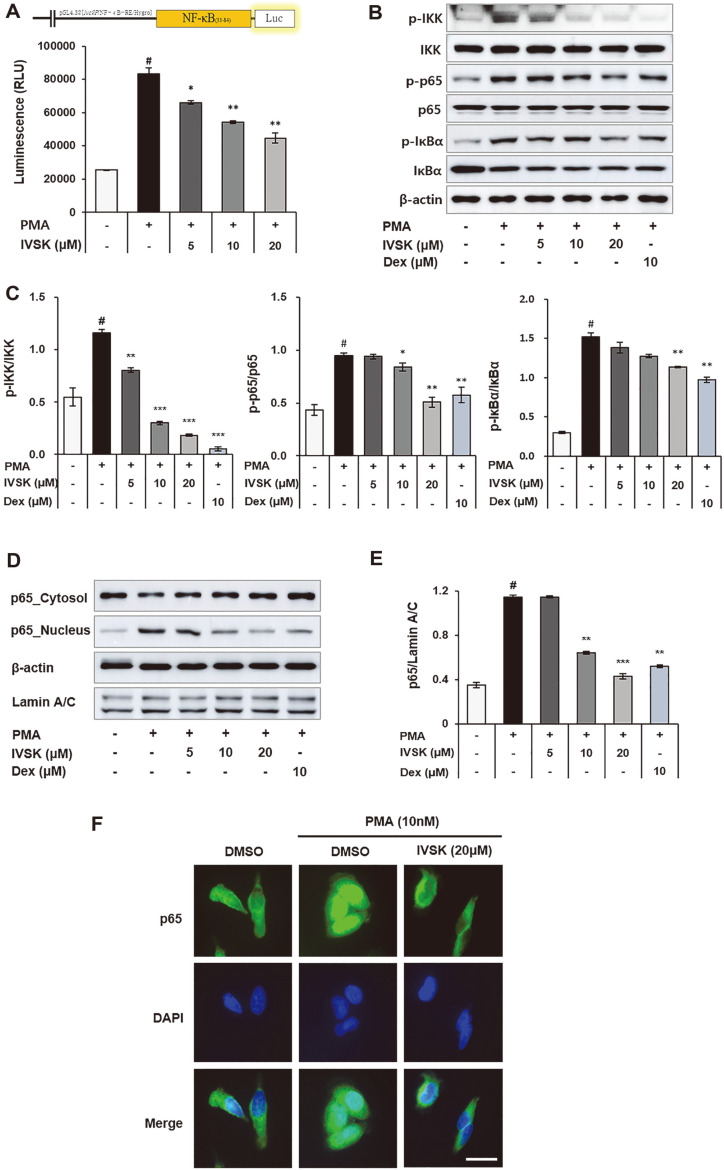
Effects of IVSK on the phosphorylation of IKK and NF-κB and the translocation of p65 in PMAstimulated A549 cells. A549 cells transfected with NF-κB were treated with IVSK at each respective concentration, and subsequently, the cells were stimulated with 100 nM PMA. The luciferase activity was measured afterwards (**A**). Phosphorylation of NF-κB p65 and IκBα (**B, C**) and nuclear translocation of NF-κB in the protein fractions (**D, E**) were evaluated using western blot analysis. Nuclear translocation of NF-κB p65 was also confirmed by immunocytochemistry (Scale bar; 100 μm) (**F**). Data are expressed as the mean ± SD. ^#^*p* < 0.05 vs. negative control group; *p < 0.05, **p < 0.01 and ***p < 0.001 vs. PMA only group.

We used A549 cells transfected with the NF-κB pGL4.32 vector. When the transfected A549 cells were treated with 100 nM PMA, the difference was more than 2 times compared to the non-treated cells. Therefore, from the result of performing a luciferase assay by treatment with 100 nM PMA, we found that the binding of NF-κB p65 to the promoter site decreased as the concentration of IVSK increased (Fig. 3A).

Generally, the activity of IKK is catalytically activated in response to various immune receptor signals. NF-κB induces nuclear translocation based on the degradation of IκBα. This path depends on the activation of the IKK complex consisting of IKKα, IKKβ and IKKγ, and the activation of IKK is affected by protein kinase C (PKC) [13]. In a standard procedure, when A549 cells were treated only with PMA, it was confirmed by western blot that the phosphorylation of IKK, p65 and IκBa increased. IVSK suppressed the phosphorylation of IKK, p65, and IκB induced by PMA (Figs. 3B and 3C). To confirm the nuclear translocation of NF-κB p65, experiments were carried out separately for cytosol and nuclear proteins. The results show that IVSK significantly inhibited the translocation of p65 (Figs. 3D and 3E). Similarly, the immunocytochemistry results show that 20 μM of IVSK inhibited the nuclear translocation of NF-κB p65 (Fig. 3F). Therefore, it could be argued that the anti-inflammatory effect of IVSK appears first through inhibition of IKK phosphorylation and then manifests through the suppression of NF-κB signaling and more specifically the inhibition of the nuclear translocation of NF-κB p65.

### Effects of IVSK on PMA-Stimulated MAPK Activation in A549 Cells

MAPK responds to a variety of extracellular and intracellular stimuli and mediates several biological processes, including cell growth and differentiation, immunity, and inflammation. The phosphorylation of MAPK, such as p38, JNK, and ERK, is also associated with inflammation. MAPK is an upstream kinase responsible for the activation of AP-1 and NF-κB [10, 23]. MAPK is phosphorylated by MAPK kinase (MMK), and MMK is phosphorylated and activated by mitogen-activated protein (MAP) kinase kinase kinase (MAP3K) [13].

**Fig. 4:**
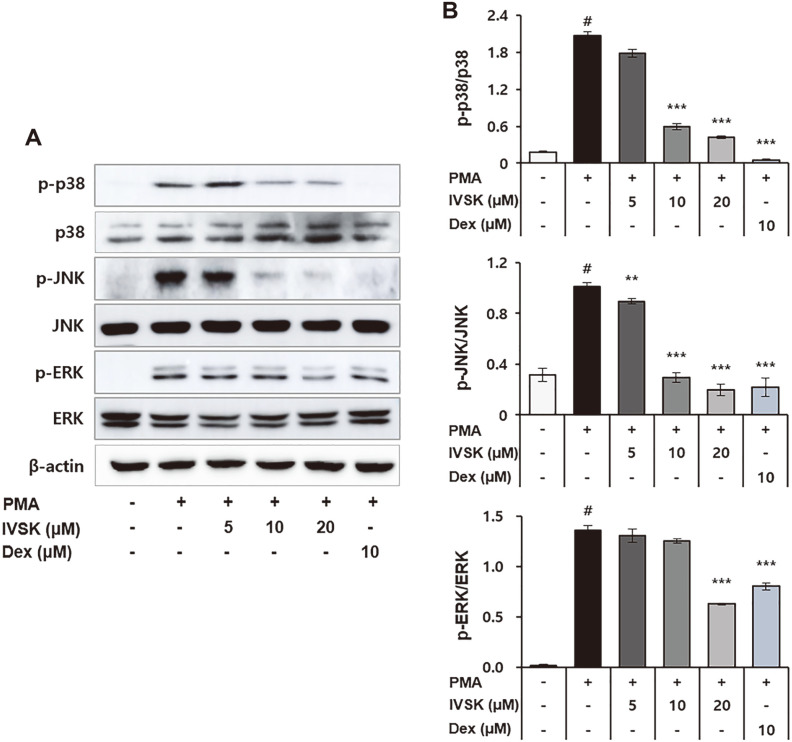
Effects of IVSK on MAPK activation in PMA-stimulated A549 cells. A549 cells were treated with IVSK and subsequently stimulated with 10 nM PMA. The phosphorylation of MAPK p38, JNK and ERK are shown (**A, B**). Data are expressed as the mean ± SD. ^#^*p* < 0.05 vs. negative control group; *p < 0.05, **p < 0.01 and ***p < 0.001 vs. PMA only group.

Consequently, we further used western blot analysis to evaluate whether IVSK would regulate PMA-induced p38, JNK, and ERK activation. The results confirmed that phosphorylation of MAPK could be upregulated by PMA treatment, but the subsequent addition of IVSK could, in turn, reduce the phosphorylation of MAPK (Figs. 4A and 4B). Therefore, it was shown that the anti-inflammatory effect of IVSK can be supported by inhibiting MAPK, which has an important role in inflammation.

### Effects of IVSK on PMA-Stimulated Transcriptional Activity and Nuclear Translocation of AP-1 in A549 Cells

**Fig. 5:**
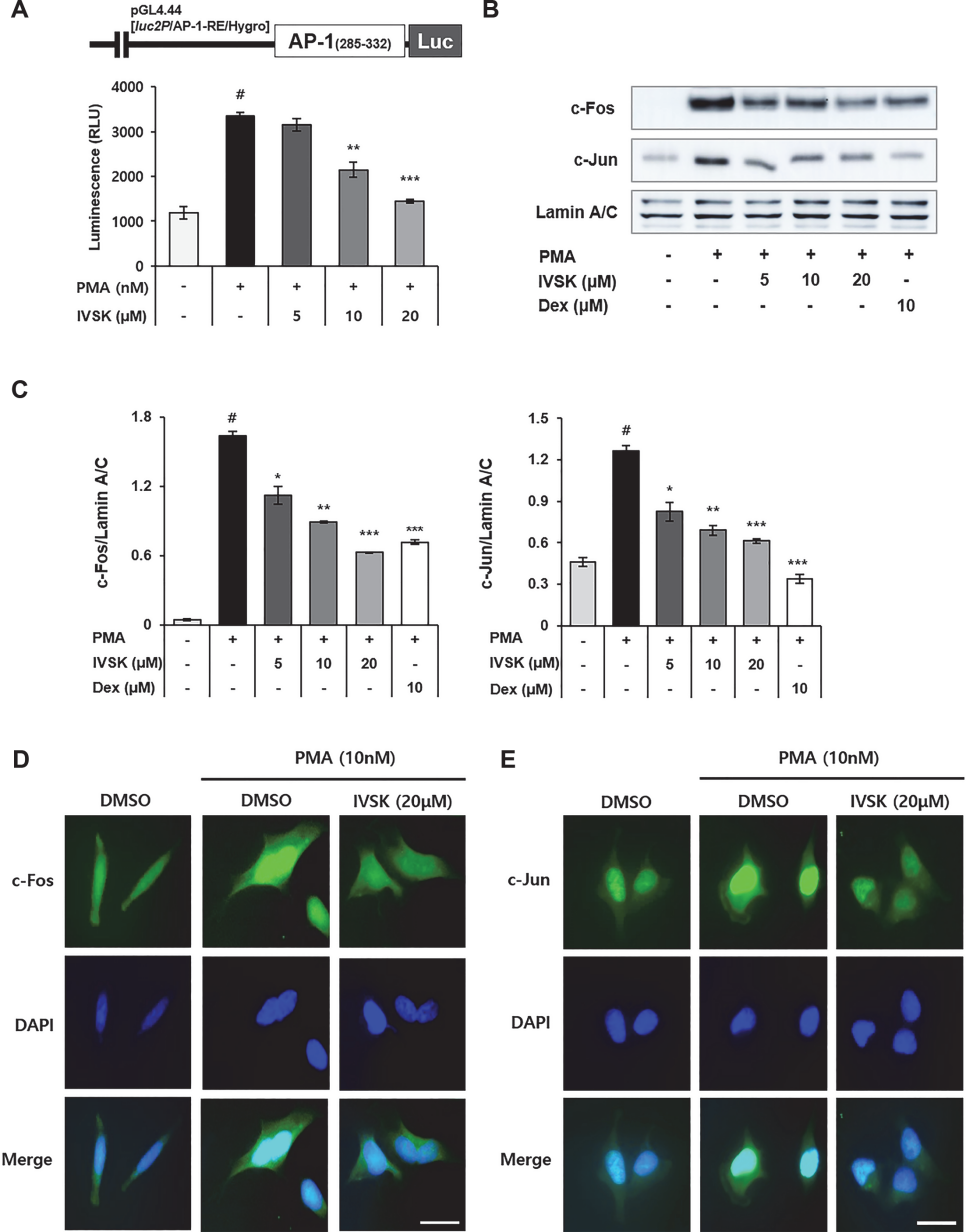
Effects of IVSK on the transcriptional activity and nuclear translocation of AP-1 in PMA-stimulated A549 cells. A549 cells were treated with IVSK and subsequently stimulated with 50 nM PMA. The luciferase activity was measured afterward (**A**). A549 cells were treated with IVSK and subsequently stimulated with 10 nM PMA. Nuclear extracts were isolated from the cells. c-Jun and c-Fos in the nuclear extracts were detected using western blotting (**B**) and normalized with Lamin A/C (**C**). Nuclear translocation of c-Fos (**D**) and c-Jun (**E**) was also confirmed by immunocytochemistry (Scale bar; 100 μm). Data are expressed as the mean ± SD. ^#^*p* < 0.05 vs. negative control group; *p < 0.05, **p < 0.01 and ***p < 0.001 vs. PMA only group.

Experiments were conducted to confirm the inhibitory effect of IVSK on AP-1, a transcription activator consisting of c-Fos and c-Jun [23], which has an important role in inflammation. We used A549 cells transfected with the AP-1 pGL4.44 vector. When the transfected A549 cells were treated with 50 nM of PMA, the difference was more than 2 times compared to the non-treated group. Therefore, 50 nM of PMA was the concentration used in the experiment. The results of the luciferase assay showed a decrease in the binding of AP-1 at the promoter site as the IVSK concentration was increased (Fig. 5A). The western blot experiments for c-Jun and c-Fos, done separately for the cytosol and nuclear fractions, showed that IVSK would significantly inhibit the translocation of c-Jun and c-Fos to the nucleus (Figs. 5B and 5C). The exact same trend was observed in the immunocytochemistry results for both c-Jun and c-Fos (Figs. 5D and 5E). Therefore, it could be claimed that the anti-inflammatory effect of IVSK also embodies the suppression of the nuclear translocation of c-Jun and c-Fos.

## Discussion

In the past, the roots of PJT were mainly used for medicinal material, however recently the use of its leaves as a food is increasing [24]. A recent study showed that the extracts of PJT are effective against asthma by inhibiting OVA-induced allergies and downregulating inflammation mediators [18]. In addition, the active materials in PJT have been separated into single compounds and are known to effectively inhibit NO [20]. However, previous studies regarding the identification of individual compounds responsible for the claimed bioactivity and their anti-inflammatory properties are limited. We measured NF-κB luciferase activity by treating 15 compounds isolated from PJT to PMA-stimulated A549, and found that IVSK potently inhibited the NF-κB activity. Therefore, we clarified the anti-inflammatory effect of IVSK and its anti-inflammation mechanism in this study. Specifically, we confirmed that IVSK has an anti-inflammatory effect by reducing the secretion of substances associated with the inflammatory reaction and effectively inhibiting NF-κB and AP-1.

We performed these experiments using A549 cells treated with PMA to artificially induce an inflammatory response. PMA is used to evaluate anti-inflammatory effects and the mechanisms of drug candidates as an in vitro model and is well known to induce the production of inflammatory mediators including cytokines and chemokines [12, 25]. Initially, PMA treatment in A549 cells activates PKC and then activates IKK which leads to the phosphorylation and degradation of IκBα [26, 27]. NF-κB is phosphorylated and then travels to the nucleus regulating the transcription of a specific target gene and inducing an inflammatory reaction [28]. Furthermore, MAPK is involved in various aspects of the inflammatory response with AP-1, which is an important transcription factor in addition to NF-κB [25, 29].

IVSK up to a concentration of 20uM was found to be non-cytotoxic with or without PMA (Figs. 1B and 1C). As a control experiment, PMA increased the inflammatory response in lung epithelial cells. However, IVSK-pretreated cells had significantly greater reduction of inflammatory mediators when compared with control, suggesting that IVSK has beneficial effects in PMA-induced inflammation. The pre-treatment of IVSK suppressed the secretion of the pro-inflammatory mediators IL-8 and MCP-1 during the PMA-induced inflammatory response (Figs. 1D-1E). In addition, the expression levels of IL-1β, IL-6, IL-8 and MCP-1 were shown to be decreased at the mRNA level (Fig. 2). This result is consistent with a previous study showing that the PJT extract is effective against OVA-induced allergic asthma [18].

Recent studies reported that cytokines and chemokines are elevated in the bronchoalveolar lavage fluid (BALF) and the sputum of asthmatic patients [30], as well as being significant for assessing phenotyping asthmatics [31]. Lung epithelial cells are a source of inflammatory cytokines such as IL-1β, IL-6, and IL-8. These interleukins can activate the immune system and contribute to acute inflammation by promoting antigen presentation, expression of matrix metalloproteins, and the development and differentiation of inflammatory cells [32]. Particularly, IL-1β upregulates the acute inflammatory response in respiratory distress syndrome by promoting the secretion of inflammatory cytokines from epithelial cells [33]. Gene expression of IL-8 is driven by cytokines, growth factors, and oxidants, which are genetically associated with asthma [34]. The resulting IL-8 is secreted and acts as a central mediator of lung damage, and a high dose of IL-8 induces bronchial hyperresponsiveness [35]. In addition, secretion of the chemoattractant cytokine MCP-1 contributes to the host defense by promoting the adsorption of inflammatory cells, including eosinophils and lung inflammation [36]. Barnes and Karin reported that the production of various inflammatory molecules is controlled by the activation of transcriptional factors through the phosphorylation of MAPK [37].

Therefore, it was suspected that the decrease in pro-inflammatory mediators is the central mechanism behind the anti-inflammatory response of IVSK. PMA is known to phosphorylate major MAPKs such as p38, JNK, and ERK [38]. Phosphorylated MAPKs affect the phosphorylation of NF-κB and AP-1 [2, 23]. In turn, inflammatory reactions are induced by the nuclear influx of NF-κB p65, c-Jun and c-Fos [39]. Consistent with our hypothesis, IVSK inhibited the phosphorylation of MAPK, which was activated by PMA in the A549 cells (Fig. 4). In addition, IVSK can effectively suppress multiple key components of the inflammatory cascade by blocking the activation of NF-κB and AP-1 (Figs. 3 and 5).

**Fig. 6:**
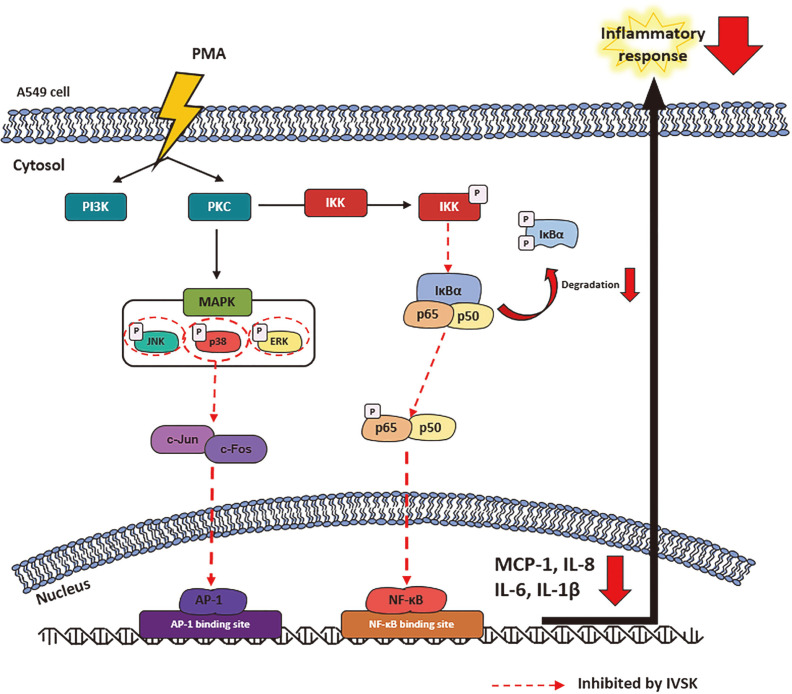
Mechanism of the anti-inflammatory effect of IVSK in PMA-stimulated A549 cells. IVSK reduced phosphorylation of IKK, IκBα, p65 and MAPK, and it inhibited the nuclear translocation of NF-κB and AP-1. Therefore, IVSK is expected to have anti-inflammatory effects. PMA, phorbol 12-myristate 13-acetate; MAPK, mitogen-activated protein kinase; JNK, c-Jun N-terminal kinase; ERK, extracellular signal-regulated kinase; AP-1, activator protein-1; IKK, IκBα kinase; NF-κB, nuclear factor-κB; MCP-1, monocyte chemoattractant protein-1.

In conclusion, based on these experimental results, it seems reasonable to claim that IVSK exhibits anti-inflammatory effects by inhibiting the phosphorylation of MAPK and the nuclear translocation of the transcription factors NF-κB and AP-1 in A549 cells stimulated with PMA (Fig. 6). Therefore, we speculate that although IVSK has an important role, the full anti-asthmatic and NO inhibitory effects of the PJT extract cannot be attributed to IVSK alone. It is necessary to identify the individual anti-inflammatory effects of the other constituents of the PJT extract and the potential synergistic relationships between them, including whether IVSK is involved. Target studies with additional molecular structures are needed to determine the main target of IVSK more accurately.

## Figures and Tables

**Table 1:** Primers for PCR.

Name		Sequence	Size
IL-6	forward	5’-GAC AGC CAC TCA CCT CTT CA-3’	124 bp
	reverse	5’-AGT GCC TCT TTG CTG CTT TC-3’	
IL-8	forward	5’-ATG ACT TCC AAG CTG GCC CTG GCT-3’	299 bp
	reverse	5’-TTA TGA ATT CTC AGC CCT CTT CAA AAA-3’	
MCP-1	forward	5’-GCT CAT AGC AGC CAC CTT CAT TC-3’	203 bp
	reverse	5’-GGA CAC TTG CTG CTG GTG ATT C-3’	
IL-1β	forward	5’-GGA CAA GCT GAG GAA GAT GC-3’	360 bp
	reverse	5’-TCT TTC AAC ACG CAG GAC AG-3’	
GAPDH	forward	5’-CGGAGTCAACGGATTTGGTCGTAT-3’	307 bp
	reverse	5’-AGCCTTCTCCATGGTGGTGAAGAC-3’	
